# Movement disorders: a brief overview from an evolutionary perspective

**DOI:** 10.1007/s00702-025-03025-8

**Published:** 2025-10-15

**Authors:** Pedro J. Garcia Ruiz

**Affiliations:** Department of Neurology, Fundacion Jimenez Diaz, Universidad Autonoma de Madrid, Avda Reyes Católicos 2, 28040 Madrid, Spain

**Keywords:** Movement disorders, Evolutionary approach, Parkinson´s disease, Huntington´s disease, Tourette syndrome

## Abstract

Evolutionary approach tries to explain several aspects of neurological diseases under the perspective of evolution. Evolutive bottle-neck may explain some aspects of neurodegenerative diseases. Additionally, a number of genetic alleles may reflect an evolutionary trade-off between benefits in early life and late-life disadvantages. Antagonistic pleiotropy theory suggests that some adaptive evolutionary changes increase individual fitness early in life, but can exacerbate detrimental aging-related processes We discuss three diseases with movement disorders from an evolutionary perspective: Parkinson’s disease, Huntington’s disease, and Tourette syndrome.

## Introduction

The evolutionary approach seeks to explain aspects of neurological diseases through the lens of evolution. Many neurological diseases affecting humans are not found in other animals (Parkinson´s disease, Alzheimer´s disease etc.) including nonhuman primates, and likely have changed over time (Nesse and Williams 1998; Rigby Dames et al. 2023).

An evolutionary bottleneck may explain some features of neurodegenerative diseases; during evolution certain subcortical regions did not expand in humans to the same extent as the neocortex, and this growth discrepancy may have placed strain on the basal ganglia (BG) (Rigby Dames et al. 2023).

Additionally, a number of genetic alleles may reflect an evolutionary trade-off between benefits in early life and late-life disadvantages (Nesse and Williams 1998; Rigby Dames et al. 2023). Antagonistic pleiotropy (AP) theory suggests that some adaptive evolutionary changes facilitate reproduction and individual fitness early in life, but can exacerbate detrimental aging-related processes (Provenzano and Deleidi 2021; Morton et al. 2019; Hashimoto et al. 2018). Perhaps one of the most extensively documented examples of AP in humans is the relatively high frequency of the E4 allele. Although strongly associated with late-onset Alzheimer’s disease (AD), carriers of this allele may have enhanced cardiovascular fitness and resistance to infection (Smith and Ashford 2023; Topriceanu et al. 2024). However, the advantages, which may extend into the reproductive period, come at the cost of increased risk for AD later in life (Smith and Ashford 2023; Topriceanu et al. 2024).

We discuss three diseases associated with movement disorders from an evolutionary perspective: Parkinson’s disease, Huntington’s disease, and Tourette syndrome.

## Parkinson’s disease

Parkinson’s disease (PD) is, at present, the second most prevalent neurodegenerative disorder (Kalia and Lang 2015; Savica et al. 2016). A central tenet of the evolutionary perspective is that PD is a uniquely human disease, not found in other non-human species (Matsuda et al. 2009; Garcia-Ruiz and Espay 2017; Diederich et al. 2019; Diederich et al. 2020). The presence of Lewy bodies and α-synuclein (α-syn) accumulation in the substantia nigra is accepted as a basic pathological signature of PD, although α-syn is not specific and contributes to multiple neurodegenerative processes (Riederer et al. 2019, 2023). In any case, the most distinct characteristic of PD pathology is the progressive loss of dopaminergic neurons within the Substantia nigra pars compacta (SNpc). (Riederer et al. 2019, 2023). Genetic component plays an important role in PD, especially in younger patients (Kalia and Lang 2015; Bloem et al. 2021; Poplawska-Domaszewicz et al. 2024).

The convergence of genetic, environmental, and behavioral factors in the development of PD suggests that evolutionary processes may partially explain the vulnerability of the human brain to this form of neurodegeneration (Garcia-Ruiz and Espay 2017; Diederich et al. 2019, 2020).

Diederich et al. (2019, 2020) suggested that the dramatic expansion of the human telencephalon, and particularly the neocortex, imposed a significant burden on subcortical circuits, especially the BG. Moreover, the expansion of the BG did not keep pace with that of the cortex, particularly in SNpc, which was pushed to its functional limit (Matsuda et al. 2009; Bolam and Pissadaki 2012; Pissadaki and Bolam 2013; Diederich et al. 2019, 2020). Several reasons explain why the SNpc is the weakest link in the BG circuit:

1. The axonal arborization of dopaminergic neurons in the SNpc can be far more extensive than those of other neurotransmitter types (Matsuda et al. 2009; Diederich et al. 2019, 2020). Anatomical studies by Matsuda et al (Matsuda et al. 2009) showed that the axon of a single tyrosine hydroxylase-positive dopaminergic neuron occupies up to 6% of the volume of the striatum, which is remarkable.
2. Dopaminergic neurons have broad action potentials and are autonomous pacemakers, exhibiting large oscillations in intracellular Ca2 + concentration driven by the opening of voltage-dependent Cav1 (L-type) Ca2 + channels (Surmeier et al. 2017; Diederich et al. 2020). Nigral dopamine neurons exhibit high basal levels of mitochondrial oxidant stress and free cytosolic Ca2+ (Bolam and Pissadaki 2012; Pissadaki and Bolam 2013; Diederich et al. 2020).
3. Finally, due to massive arborization, human mesostriatal neurons occupy a much larger volume of the striatum than those of other vertebrates. Bolam and Pissadakis (Bolam and Pissadaki 2012; Pissadaki and Bolam 2013) highlighted the pronounced expansion of these neurons into the striatum compared with other mammals. Human nigral dopaminergic neurons must give rise to axons that are ten times the size and ten times the number of synapses in rats (Matsuda et al. 2009; Bolam and Pissadaki 2012; Pissadaki and Bolam 2013). According to the authors, this axonal architecture creates high energy demands on dopamine-producing nigral neurons to maintain cell function (Bolam and Pissadaki 2012; Pissadaki and Bolam 2013).

In summary, nigral dopaminergic neurons are on the edge of energetic catastrophe (Matsuda et al. 2009; Bolam and Pissadaki 2012; Pissadaki and Bolam 2013; Diederich et al. 2019, 2020; Surmeier et al. 2017). An evolutionary bottleneck was reached with the expansion of the massive, unmyelinated nigrostriatal axonal arborization (Matsuda et al. 2009; Bolam and Pissadaki 2012; Pissadaki and Bolam 2013). This peculiar nigral overload and the neurophysiological characteristics of these neurons may partly explain the selective fragility of human dopaminergic mesencephalic neurotransmission and the unique presence of PD in humans (Bolam and Pissadaki 2012; Pissadaki and Bolam 2013; Garcia-Ruiz and Espay 2017; Diederich et al. 2019; Diederich et al. 2020; Rigby Dames et al. 2023; Surmeier et al. 2017).

Another compelling evolutionary aspect of PD concerns common genetic mutations associated with the disorder. Mutations in the *LRRK2* gene are the most frequent cause of familial PD (Kalia and Lang 2015; Bloem et al. 2021). LRRK2 is highly expressed in immune cells, and this expression is tightly regulated by immune stimulation (Gardet et al. 2010; Wallings and Tansey 2019). The pathogenic G2019S variant is the most prevalent LRRK2 mutation found in Southern European and North African populations (Kalia and Lang 2015; Bloem et al. 2021). Provenzano and Deleidi (Provenzano and Deleidi 2021) suggested that this mutation mat confer benefits in the defense against pathogens and may have undergone positive selection in hominid evolution. AP may explain, in part, the persistence of this mutation.

## Huntington’s disease

Huntington’s disease (HD) is a devastating autosomal dominant neurodegenerative disorder characterized by abnormal movements, cognitive decline, and psychiatric disturbance (Ghosh and Tabrizi 2018; McColgan and Tabrizi 2018). The motor manifestations of HD are heterogeneous and comprise various hyperkinetic features including chorea, tics, and dystonia, which can coexist with an akinetic-rigid syndrome, typically increasing in severity over time (García Ruiz et al. 2002; van Vugt et al. 2004; Ghosh and Tabrizi 2018; McColgan and Tabrizi 2018).

HD is caused by an abnormal expansion of cytosine-adenine-guanine (CAG) trinucleotide repeats in the huntingtin gene (Gusella et al. 1993). Affected individuals carry > 39 CAG repeats, compared with fewer than 36 in healthy individuals (Gusella et al. 1993; Ghosh and Tabrizi 2018; McColgan and Tabrizi 2018; Barron et al. 2021). Those individuals with CAG repeats in the intermediate zone (36–39) may or may not develop clinical HD during their lifetime but can pass the mutation on to successive generations (Ghosh and Tabrizi 2018; McColgan and Tabrizi 2018; Barron et al. 2021). The huntingtin gene encodes the large protein huntingtin (HTT), which plays essential roles in central nervous system development, maintenance, and trafficking, including the regulation and transport of brain-derived neurotrophic factor (BDNF) (Ghosh and Tabrizi 2018; McColgan and Tabrizi 2018; Barron et al. 2021). At present, the most widely accepted mechanism of HD is the toxic effect of mutated huntingtin (Ghosh and Tabrizi 2018; McColgan and Tabrizi 2018), although loss of wild-type huntingtin has also been considered a factor (Barron et al. 2021; Ghosh and Tabrizi 2018; McColgan and Tabrizi 2018).

Given the severe effects of HD, it seems difficult to find any evolutionary benefit of this mutation. However, certain aspects of the AP hypothesis may explain some contradictory and striking features of the disease.

Van der Plas et al proposed that triplet repeat-expansion diseases represent the pathological extreme of a more general mutational process that contributes to normal brain function (van der Plas et al. 2020). According to this hypothesis, increased triplet repeats below the disease threshold could confer advantages, such that higher repeats would lead to improved brain function (Fondon et al. 2008; van der Plas et al. 2020). The Kids-HD study (Lee et al. 2018; van der Plas et al. 2020; Neema et al. 2024), a long-term prospective analysis of very young subjects (< 20 years of age) at risk for HD and free from manifestations of the disease, investigated the impact of HD mutation on brain structure and functional development several decades before the expected motor onset. Participants underwent brain imaging, cognitive assessments, and motor testing (van der Plas et al. 2020; Lee et al. 2018; Neema et al. 2024). Notably, individuals with gene expansion had significantly better cognitive, behavioral, and motor scores than non-expanded controls, along with larger cerebral volumes and cortical features. This initial peak was followed by a prolonged decline in both functional and structural measures (Neema et al. 2024), consistent with accelerated aging process (Lee et al. 2018; van der Plas et al. 2020; Neema et al. 2024). According to this fascinating hypothesis, HD carriers might have several advantages early in life, until de reproductive age, at the cost of devastating brain dysfunction late in life. This interesting hypothesis has been also suggested based on animal models of HD (Morton et al. 2019).

## Tourette syndrome

Tourette syndrome (TS) is chronic hereditary neurodevelopmental disorder characterized by multiple tics, including vocal tics, and neuropsychiatric comorbidities such as obsessive-compulsive disorder and attention-deficit disorder (Johnson et al. 2023). TS typically manifests in school-aged children and, although often not severe, a subset of patients develop disabling symptoms (Johnson et al. 2023; Baizabal-Carvallo et al. 2024). While TS poses social challenges for many patients, some individuals with the condition have exceptional abilities (Sacks 1992; Reser 2017).

Reser (Reser 2017) suggested that the clinical manifestations of tic disorders may be associated with certain adaptive advantages in specific environments. On average, individuals with TS perform behavioral tests of cognitive motor control more quickly and accurately than controls (Jackson et al. 2011).

Reser (Reser 2017) also proposed that genes linked to TS may have been naturally selected due to their role in the development of rapid physical responsiveness. It therefore seems that an evolutionary trade-off between advantageous and disadvantageous traits may play a role in TS (Sacks 1992; Reser 2017; Jackson et al. 2011).

## Conclusion

The evolutionary approach may explain certain characteristics of neurological diseases. In some cases, an evolutionary bottleneck may offer insight on conditions including PD. On the other hand, the AP theory suggests that particular adaptive evolutionary changes facilitate reproduction and individual fitness early in life, but can become detrimental with age. As a result, the theory may shed light on recent findings in PD and HD. Finally, a trade-off between the advantages and disadvantages may play a role in TS.

## Data Availability

Not applicable.
